# Supplementary figures and images for: Deciphering novel TCF4-driven mechanisms underlying a common triplet repeat expansion-mediated disease
Source: PLoS Genet. 2024 May 7;20(5):e1011230. doi: 10.1371/journal.pgen.1011230 (PMC11101122; doi:10.1371/journal.pgen.1011230)

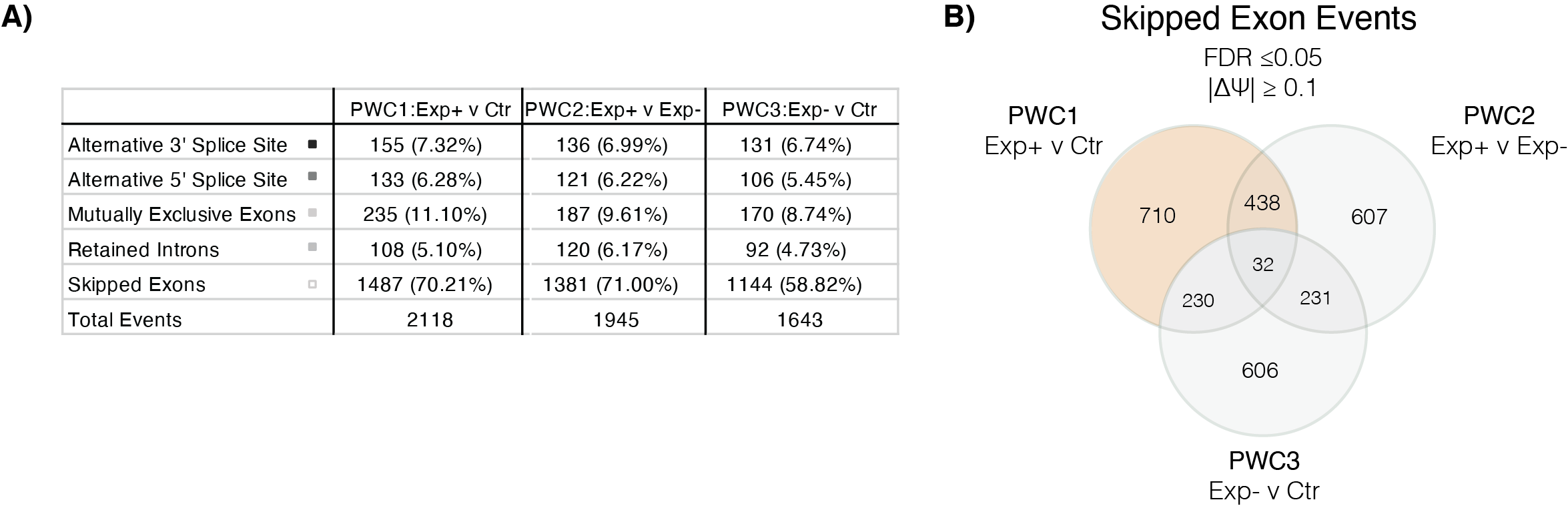

Supplement: S1 Fig — Short-read RNA-seq data generated from primary corneal endothelial cells were analysed by rMATS. A) Table of rMATS events for each pairwise comparison (PWC). Significance denoted by FDR ≤ 0.05 and deltapsi magnitude larger than 0.1. Values in brackets show percentage of total splice events B) Venn diagram of skipped exon event coordinates between all PWCs. The largest overlap is observed between PWC1 and PWC2 highlighting an enrichment of skipped exon events in Exp+ FECD compared to Exp- FECD and controls. (PNG) [file pgen.1011230.s001.png]

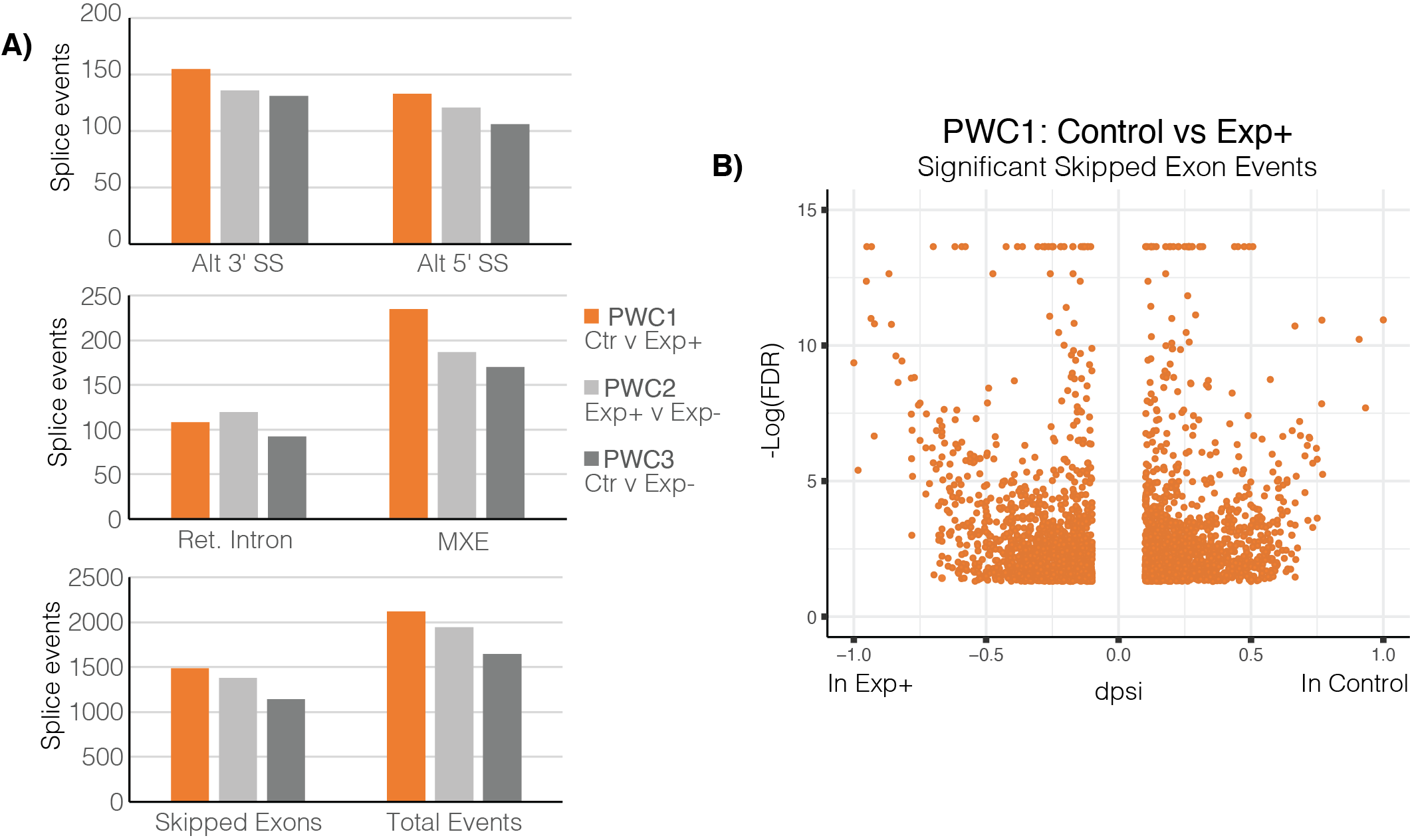

Supplement: S2 Fig — A) Summary of global differences in splicing events categorised by rMATS. N = 4 for controls, N = 3 each for FECD Exp+ and FECD Exp-. The most common splice category of alternative splicing observed between all pairwise comparisons was skipped exon, representing ~70% of all significant alternative splicing events detected. Alt 3’ SS: alternative 3’ splice site, Alt 5’ SS: alternative 5’ splice site, Ret. Intron: retained intron, MXE: mutually exclusive exon B) Volcano plot of statistically significant skipped exon events in PWC1 (Control vs Exp+). The dpsi value denotes the magnitude of change for each dysregulated skipped exon event identified. A positive dspi denotes decreased levels of exon inclusion in Exp+, whereas a negative dspi denotes increased levels of exon inclusion in Exp+. (PNG) [file pgen.1011230.s002.png]

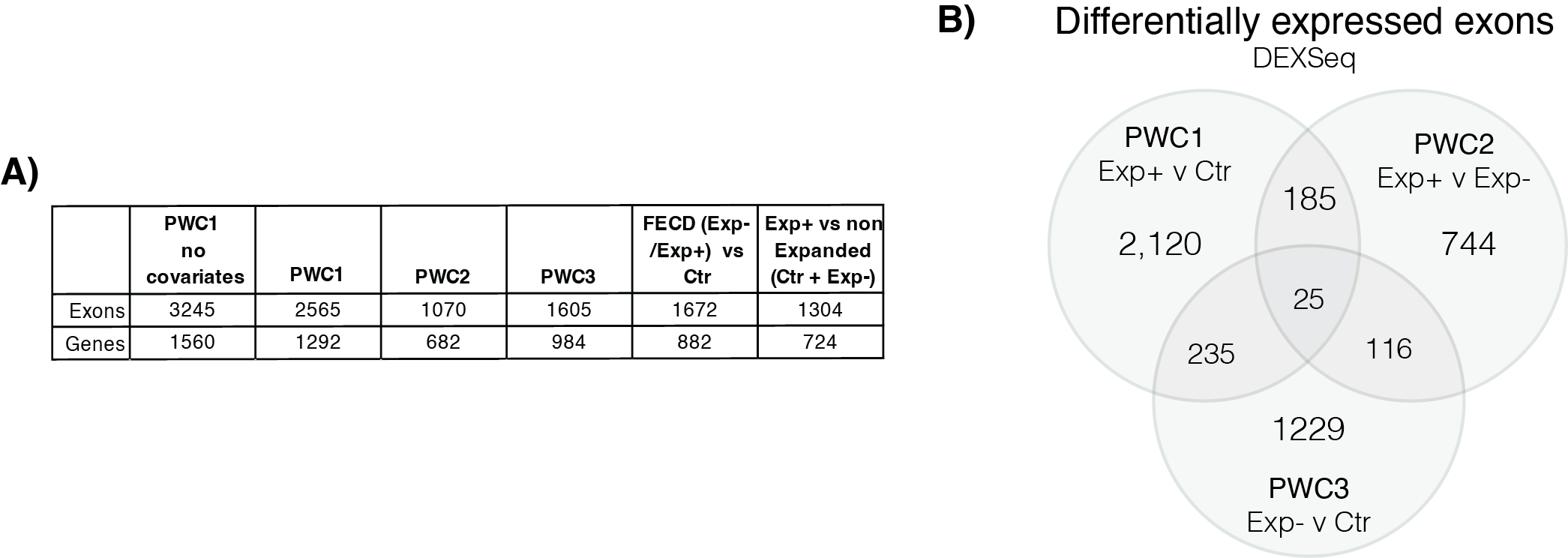

Supplement: S3 Fig — A) Table of DEXSeq2 events and genes for each pairwise comparison (PWC). Significance denoted by p-adj ≤ 0.05. B) Venn diagram of dysregulated exons coordinates between all three PWCs. The largest overlap is observed between PWC1 and PWC2, highlighting an enrichment of skipped exon events in Exp+ FECD compared to Exp- FECD and controls. (PNG) [file pgen.1011230.s003.png]
