# Supplementary material for: Deciphering novel TCF4-driven mechanisms underlying a common triplet repeat expansion-mediated disease
Source: PLoS Genet. 2024 May 7;20(5):e1011230. doi: 10.1371/journal.pgen.1011230 (PMC11101122; doi:10.1371/journal.pgen.1011230)
Supplement: S1 Table — (DOCX) [file pgen.1011230.s004.docx]

**Table S1: Clinical details of individuals with Fuchs endothelial corneal dystrophy used to established primary corneal endothelial cell cultures used for downstream analysis.**

| **Name/Sex** | **CTG18.1 genotype** | **Age at first keratoplasty (years)*** | **CCT (μm)**  **before surgery** | **Pre-op**  **BCVA** | **CCT (μm)**  **after surgery** | **Final BCVA**** | **Associated ocular features** | **Family history** |
| --- | --- | --- | --- | --- | --- | --- | --- | --- |
| FECD1exp+/F | 43/90 | 72 | NR RE  591 LE | 6/24 RE  6/12 LE | 553 RE  NS LE | 6/9 RE  6/7.6 LE | Cataract BE  ERM peel RE | NK |
| FECD2exp+/M | 14/80 | 78 | 647 RE  640 LE | 6/24 RE  6/36 LE | NS RE  NR LE | 6/7.5 RE  6/9 LE | Cataract BE | NK |
| FECD3exp+/F | 18/86 | 69 | 664 RE  NR LE | 6/9 RE  NR LE | 548 RE  565 LE**** | 6/6 RE  6/9 LE | Cataract BE | NK |
| FECD4exp+/F | 26/78 | 45 | 686 RE  720 LE | 6/5 RE  6/9 LE | NS RE  590 LE | 6/5 RE  6/5 LE | Nil | NK |
| FECD5exp+/F | 16/86 | 67 | 666 RE  681 LE | 6/9 RE  6/18 LE | NR RE  NR LE | 6/6 RE  6/6 LE | Cataract BE | NK |
| FECD6exp+/M | 12/106 | 69 | 620 RE  624 LE | 6/6 RE  6/7.5 LE | 485 RE  489 LE | 6/5 RE  6/4 LE | Cataract BE | 1 sibling |
| FECD7exp+/F | 16/93 | 54 | 633 RE  606 LE | 6/9 RE  7/7.5 LE | 515 RE  508 LE | 6/6 RE  6/5 LE | Cataract BE | NK |
| FECD8exp+/F | 63/88 | 73 | 605 RE  630 LE | 6/18 RE  6/6 LE | 513 RE  NS LE | 6/5 RE  6/6 LE | Cataract BE | NK |
| FECD9exp+/M | 12/76 | 54 | 543 RE  636 LE | RE 6/9  LE 6/12 | NS RE  NR LE | 6/7.5 RE  6/6 LE | Cataract BE | NK |
| FECD10exp+/M | 18/67 | 65 | 587 RE  791 LE | 6/9 RE  6/9 LE | NR RE  NR LE | 6/6 RE  6.7.5 LE | Cataract BE | NK |
| FECD1exp-/F | 13/18 | 61 | 599 RE  588 LE | 6/24 RE  6/9 LE | 690 RE****  NR LE | 6/6 RE  6/9 LE | Cataract BE | NK |
| FECD2exp-/F | 18/24 | 71 | NR RE  699 LE | 6/12 RE  6/12 LE | 7151RE****  572 LE | 6/6 RE  6/5 LE | Cataract BE | NK |
| FECD3exp-/M | 22/28 | 53 | 567 RE  564 LE | 6/12 RE  6/6 LE | 503 RE  NS LE | RE 6/5  LE 6/6 | Cataract BE | NK |
| FECD4exp-/F | 15/15 | 48 | 553 RE  524 LE | 6/18 RE  6/9 LE | 512 RE  498 LE | 6/5 RE  6/5 LE | Cataract BE | NK |
| FECD5exp-/F | 16/23 | 56 | 605 RE  589 LE | 6/12 RE  6/12 LE | 512 RE  504 LE | 6/6 RE  6/6/ LE | Cataract BE | NK |
| FECD6exp-/F | 15/16 | 58 | NR RE  NR LE | 6/9  6/12 | 535 RE  526 LE | 6/7.5 RE  6/7.5 LE | Cataract BE | NK |

M male, F female, CCT central corneal thickness, BCVA best corrected visual acuity, NR no record, NK Not known, NS No surgery performed, RE right eye, LE left eye, BE both eyes, *Descemet membrane endothelial keratoplasty (DMEK) unless stated, **BCVA best corrected visual acuity with spectacle correction (Snellen annotation), **** Descemet stripping automated endothelial keratoplasty (DSAEK), ERM epiretinal membrane
