# Supplementary material for: Deciphering novel TCF4-driven mechanisms underlying a common triplet repeat expansion-mediated disease
Source: PLoS Genet. 2024 May 7;20(5):e1011230. doi: 10.1371/journal.pgen.1011230 (PMC11101122; doi:10.1371/journal.pgen.1011230)
Supplement: S3 Table — In depth characterization of isoforms in control and Exp+ long-read RNA-seq utilising SQANTI3 pipeline following stringent quality control. (DOCX) [file pgen.1011230.s006.docx]

**S3 Table. Summary of SQANTI3 analysis of IsoSeq data.** In depth characterization of isoforms in control and Exp+ long-read RNA-seq utilising SQANTI3 pipeline following stringent quality control.

| **Characterization of transcripts based on splice junctions** | | | | |
| --- | --- | --- | --- | --- |
|  | Control | | Exp+ FECD | |
|  | # isoforms | # genes | # isoforms | # genes |
| Full Splice Match (FSM) | 1,163 | 1,047 | 3,130 | 2,598 |
| Incomplete Splice Match | 647 | 535 | 2,268 | 1,377 |
| Novel In Catalog (NIC) | 829 | 685 | 1,797 | 1,336 |
| Novel Not In Catalog (NNC) | 437 | 375 | 778 | 639 |
| Genic Genomic | 3 | 3 | 5 | 5 |
| Antisense | 6 | 5 | 4 | 4 |
| Fusion | 24 | 21 | 35 | 33 |
| Intergenic | 4 | 4 | 6 | 6 |
| Terminology based on Tardaguila et al 2018.[1] n= 4 for controls and n=3 for FECD Exp+ (with one sample duplicated to acquire additional depth). FSM: full splice match. ISM: Incomplete splice match. NIC: Novel in catalog. NNC: Novel not in catalog | | | | |

**References**

1. Tardaguila M, Fuente L de la, Marti C, Pereira C, Pardo-Palacios FJ, Risco H del, et al. SQANTI: extensive characterization of long-read transcript sequences for quality control in full-length transcriptome identification and quantification. Genome Res. 2018 Jan 3;28(3):396–411.
