# Supplementary material for: Deciphering novel TCF4-driven mechanisms underlying a common triplet repeat expansion-mediated disease
Source: PLoS Genet. 2024 May 7;20(5):e1011230. doi: 10.1371/journal.pgen.1011230 (PMC11101122; doi:10.1371/journal.pgen.1011230)
Supplement: S5 Table — (DOCX) [file pgen.1011230.s008.docx]

**Table S5. rMATS identified significant differentially spliced events in Exp+ matching published events with strong association to CTG18.1-expansion mediated FECD.**

| **Gene** | **Identified by rMATS (yes/no)** | **rMATS splice type** | **Differential splicing detected via SQANTI3** | **Skipped Exon Coordinates (hg38)** | **dpsi** | **FDR** |
| --- | --- | --- | --- | --- | --- | --- |
| *ABI1^3,6^* | Yes | SE | Yes | chr10:26771075 -26771089 | 0.28 | 1.82E-07 |
| *ADD3^3,6^* | No | - | Yes | - | - | - |
| *AKAP13^3,6^* | Yes | SE | Yes | chr15:85658537 -85658590 | 0.203 | 0.003372 |
| *TSPOAP1^3,6^* | Yes | SE | Excluded | chr17:58308541 -58309380 | 0.222 | 0.028978 |
| *CD46^3,6^* | No | - | Yes | - | - | - |
| *CLASP1^3,6^* | Yes | SE | Yes | chr2:121445449 -121445496 | 0.451 | 0 |
| *COPZ2^3,6^* | Yes | SE | No | chr17:48027676 -48027789 | 0.118 | 7.49E-13 |
| *EXOC1†^3,6^* | Yes | SE | Yes | chr4:55888888 -55888932 | -0.141 | 0.000691 |
| *FGFR1†^3,6^* | Yes | SE | Yes | chr8:38429682 -38429948 | -0.248 | 0 |
| *GOLGA2^3,6^* | Yes | SE | Yes | chr9:128272785 -128272865 | -0.188 | 1.10E-07 |
| *INF2†^3,6^* | Yes | SE | Yes | chr14:104715284 -104715340 | 0.507 | 0 |
| *ITGA6†^3,6^* | Yes | SE | No | chr2:172501772 -172501901 | 0.263 | 0 |
| *KIF13A^a3,6^* | Yes | SE | Excluded | chr6:17789872 -17789910 | 0.17 | 0.00714 |
| *KIF13A^b3,6^* | No | - | Excluded | - | - | - |
| *MBNL1*^3,5,6^* | Yes | SE | Yes | chr3:152446704 -152446757 | -0.15 | 0.000337 |
| *MBNL2*^3,5,6^* | Yes | SE | Yes | chr13:97356796 -97356849 | -0.145 | 3.66E-13 |
| *MYO6^3,6^* | No | - | Yes | - | - | - |
| *NHSL1^3,6^* | Yes | SE | Yes | chr6:138441983 -138442114 | -0.222 | 0.011795 |
| *NUMA1*^3,5,6^* | Yes | SE | Yes | chr11:72012401 -72012442 | 0.268 | 0 |
| *PLEKHM2^3,6^* | Yes | SE | Yes | chr1:15721329 -15721388 | 0.234 | 1.27E-10 |
| *PPFIBP1** | Yes | SE | Yes | chr12:27677064 -27677096 | 0.162 | 7.22E-05 |
| *SCARB1^3,6^* | No | - | Yes | - | - | - |
| *SYNE1*^3,6^* | Yes | SE | Yes | chr6:152145487 -152145555 | 0.223 | 4.36E-09 |
| *VEGFA^3,6^* | No | - | Yes | - | - | - |
| * Events previously validated by RT-PCR, in addition to Mixture of Isoforms (MISO) and MAP-RSeq.^6–8^  † Events where the dysregulated exons match published work, but the flanking exons are not a perfect match.  SE: skipped exon. Samples labelled as excluded from Iso-seq analysis were excluded due to insufficient coverage during sequencing | | | | | | |
