# Supplementary material for: Deciphering novel TCF4-driven mechanisms underlying a common triplet repeat expansion-mediated disease
Source: PLoS Genet. 2024 May 7;20(5):e1011230. doi: 10.1371/journal.pgen.1011230 (PMC11101122; doi:10.1371/journal.pgen.1011230)
Supplement: S6 Table — (DOCX) [file pgen.1011230.s009.docx]

**Table S6: Details on the pairwise comparison(s) where rMATS identified new significant differentially spliced events matching published differentially spliced genes with strong association to CTG18.1-expansion mediated FECD.**

| **Gene** | **rMATS splice type** | **Exon Coordinates (hg38)** | **FDR** | **dpsi** | **Verified in Iso-Seq** |
| --- | --- | --- | --- | --- | --- |
| *FGFR1* | MXE | chr8:38429681-38429948, 38457355-38457534 | 0 | 0.125 | Yes |
| *MBNL1* | SE | chr3:1522994040-152300367 | 1.13E-10 | -0.101 | Yes |
| *KIF13A* | SE | chr6:17789871-17789910 | 1.73E-09 | 0.339 | Excluded |
| *KIF13A* | SE | chr6:17794248-17794395 | 1.63E-07 | 0.125 | Excluded |
| *AKAP13* | SE | chr15:85662387-85662453 | 0.0012 | 0.126 | Yes |
| *MBNL2* | SE | chr13:97356795-97356849 | 0.0492 | -0.304 | Yes |
| *MBNL2* | SE | chr13:97366458-97366553 | 0.0011 | -0.114 | Yes |
| *NUMA1* | A5SS | chr11:72068078-72068231 (long), 72068104-72068231 (short) | 0.0441 | 0.113 | No |
| *NUMA1* | SE | chr11:72049418-72049539 | 0.0175 | -0.107 | Yes |
| *TSPOAP1* | SE | chr17:58320108-58320129 | 0.0002 | -0.362 | Excluded |
| MXE: mutually exclusive exon event. SE: skipped exon event. A5SS: alternative 5’ splice site event. Genes excluded from Iso-Seq analysis were done so due to insufficient coverage | | | | | |
