# Supplementary material for: Deciphering novel TCF4-driven mechanisms underlying a common triplet repeat expansion-mediated disease
Source: PLoS Genet. 2024 May 7;20(5):e1011230. doi: 10.1371/journal.pgen.1011230 (PMC11101122; doi:10.1371/journal.pgen.1011230)
Supplement: S7 Table — (DOCX) [file pgen.1011230.s010.docx]

**Table S7: Differential gene expression and alternative splicing of fibronectin (*FN1*) in all three pairwise comparisons.**

| **Differential gene expression (DESeq2)** | | | |
| --- | --- | --- | --- |
|  | *shrunkLFC* | *padj* | |
| PWC1 | 2.96419441 | 3.20x10^-20^ | |
| PWC2 | n.s | n.s | |
| PWC3 | 1.9315629 | 3.37x10^-08^ | |
| **Alternative splicing (rMATS)** | | | |
|  | *Dysregulated exons* | *dPSI* | *FDR* |
| PWC1 | Exon 25 (EDB)  Exon 33 (EDA) | -0.253,  -0.305 | 0,  0 |
| PWC2 | Exon 25 (EDB)  Exon 33 (EDA) | 0.262,  0.179 | 0,  4.76E-05 |
| PWC3 | Exon 25 (EDB) | -0.126 | 0.007044931 |
| Exon 25 (EDB) is defined by the genomic coordinates chr2:215,392,931-215,393,203 and Exon 33 (EDA) by chr2:215,380,811-215,381,080. Padj = FDR-adjusted p-value, dPSI=delta percent spliced in, FDR = False discovery rate, n.s. = not significant | | | |
