# Supplementary material for: Deciphering novel TCF4-driven mechanisms underlying a common triplet repeat expansion-mediated disease
Source: PLoS Genet. 2024 May 7;20(5):e1011230. doi: 10.1371/journal.pgen.1011230 (PMC11101122; doi:10.1371/journal.pgen.1011230)
Supplement: S8 Table — (DOCX) [file pgen.1011230.s011.docx]

**Table S8: rMAPS-identified RNAbinding motif enrichment in PWC1 and PWC2, which was also absent in PWC3.**

| **GC-containing motifs upregulating skipped exon in Exp+** | |
| --- | --- |
| PCBP2 | CC[CT][CT]CC[ACT} |
| RBM4 | GCGCG[GC][GC] |
| RBM4 | GCGCG[GC]G |
| RBM45 | GACGA[AC][ACG] |
| **TT-containing motifs downregulating skipped exon events in Exp+** | |
| HNRNPC | [ACT]TTTTT[GT] |
| HNRNPCL1 | [ACT]TTTTT[GT] |
| PCBP1 | C[CT]TTCC |
| ZC3H14 | TTT[AGT]TTT |
