# Supplementary material for: Deciphering novel TCF4-driven mechanisms underlying a common triplet repeat expansion-mediated disease
Source: PLoS Genet. 2024 May 7;20(5):e1011230. doi: 10.1371/journal.pgen.1011230 (PMC11101122; doi:10.1371/journal.pgen.1011230)
Supplement: S11 Table — (DOCX) [file pgen.1011230.s014.docx]

**Table S11: Dysregulated RNA binding proteins (RBPs) uniquely in PWC1.**

| **Downregulated uniquely in Exp+ (n=35)** |
| --- |
| *REXO5,* ***ESRP2****, ZNF106, CPEB3,* ***DDX25****, ENDOU, IFIH1, WARS2, ZNFX1, MRPS7, MIF4GD, RPS4Y1, VARS2,* ***DDX60****, PNPT1, SCAF11, RRNAD1, PARP1, AFF3, PDCD4, TRMT44, MOV10, PPARGC1B, CELF5, ZNF385A, RAVER2, RBM47, DIS3L, CSDC2, NSUN7, PLD6, DDX60L, IFIT1, ELAVL3, NYNRIN* |
| **Upregulated uniquely in Exp+ (n=148)** |
| *MSL3, SAMD4A, STAU2, TPR, R3HDM1, RRP12, YBX3, CCAR1, U2AF2, IPO5, AFF4, MBNL3, BZW1, GEMIN5, TUT7, AARS1, LRRFIP2,* ***HNRNPM****, ZC3H14, RBM3, PARP4, GSPT1, POP1,* ***DDX49****, GARS1, EIF3B, UTP6, GAR1, CCDC86, CPSF6, HINT3, RRP9, EIF4G1, PNO1, FARSB, RPF1, PRDX1, HEATR1, PTBP3, ENOX1, UTP20, ZC3H13, SPATS2, XPO5, RIOK1, ATXN1, DGCR8, EIF5A, SYNCRIP, CAPRIN1, IGF2BP3, BZW2, EPRS1, RANBP6, RTCA, SSB, LARP1B, HNRNPA1L2, WARS1, SERBP1, ISG20L2, PNLDC1, NONO, PDCD11,* ***CELF1****, INTS4,* ***MBNL1****, PELO, LARP1, SCAF4, UTP14A, RBPMS, RRP1, ZNF326, DCAF13, NOL6,* ***DDX21****, TRMT61A, URM1, XPO6, RNASEH1, ISG20, MRPL52, AGFG1, CTU2, SRP72, CAVIN1, RRS1, FARSA, NOP10, CNOT10, PCBP3, XPOT, NOC2L, SF3B3, IARS1, YRDC, IPO4, DYNC1H1, TOP1, ARHGEF28, ZNF579, RBM14, EIF6, NA, BOP1* |
