# Supplementary material for: Deciphering novel TCF4-driven mechanisms underlying a common triplet repeat expansion-mediated disease
Source: PLoS Genet. 2024 May 7;20(5):e1011230. doi: 10.1371/journal.pgen.1011230 (PMC11101122; doi:10.1371/journal.pgen.1011230)
Supplement: S12 Table — (DOCX) [file pgen.1011230.s015.docx]

**Table S12: Summary of all *TCF4* DEXSeq runs**

|  | **PWC1**  **(Exp+ vs Ctr) no covariates** | **PWC1**  **(Exp+ vs Ctr) covariates** | **PWC2**  **(Exp+ vs Exp-)**  **covariates** | **PWC3**  **(Ctr vs Exp-)**  **covariates** | **Exp+ vs non-Expanded**  **(Ctr + Exp-)**  **covariates** | **FECD**  **(Exp-/Exp+ vs Ctr)**  **covariates** |
| --- | --- | --- | --- | --- | --- | --- |
| **Upregulated exons** | E108, E109, E114 | E108, E105 | E013, E014, E015, E083, E084, E108, E109, E110 | n.s | E108, E109 | n.s |
| **Upregulated isoforms** | 6 out of 93 isoforms | 3 out of 93 isoforms | 31 out of 93 isoforms | -- | 4 out of 93 isoforms | -- |
| **Downregulated exons** | E118, E119, E139, E157, E158, | n.s. | E081, E082, E117, E118, E119, E139, E140, E142, E143, E144, E158 | n.s | E119, E157, E158 | n.s |
| **Downregulated isoforms** | 37 out of 93 isoforms | -- | 73 out of 93 isoforms | -- | 63 out of 93 isoforms | -- |
| **Intron retention** | E173, E174 | n.a. |  | n.a. |  | n.a. |
