# Supplementary material for: Deciphering novel TCF4-driven mechanisms underlying a common triplet repeat expansion-mediated disease
Source: PLoS Genet. 2024 May 7;20(5):e1011230. doi: 10.1371/journal.pgen.1011230 (PMC11101122; doi:10.1371/journal.pgen.1011230)
Supplement: S14 Table — (DOCX) [file pgen.1011230.s017.docx]

**Table S14:** **rMATS *TCF4* mutually exclusive exon events demonstrating a shift between longer isoforms containing 2+ AD domains and shorter isoforms containing 1-2 AD domains detected via alternative splicing pipelines.**

|  | **NM_001083962.2/ENST00000354452.8 exon numbering** | |  |  |
| --- | --- | --- | --- | --- |
| **Splice type event (PWC1)** | **Exon 7** | **Exon 6** | **dpsi** | **FDR** |
| Mutually exclusive exon | chr18:55350873-55351003 | chr18:55403453-55403518 | 0.103 | 0.0023 |
| Mutually exclusive exon | chr18:55350873-55351000 | chr18:55403453-55403518 | 0.102 | 0.0030 |
| **Consequence:** | **Higher in Exp+** | **Higher in Control** |  |  |
|  | **First exon common to both long and short isoforms** | **Last exon common to the majority of long isoforms** |  |  |
