# Supplementary material for: Deciphering novel TCF4-driven mechanisms underlying a common triplet repeat expansion-mediated disease
Source: PLoS Genet. 2024 May 7;20(5):e1011230. doi: 10.1371/journal.pgen.1011230 (PMC11101122; doi:10.1371/journal.pgen.1011230)
Supplement: S15 Table — (DOCX) [file pgen.1011230.s018.docx]

**Table S15**: ***TCF4* isoform RNAScope sample summary with results of FISH with probe targeting repeat and negative RNAScope control experiments (with probes targeting bacterial genes).**

| **Sample** | **CTG18.1 repeat genotype** | **CUG**  **specific**  **foci present** | **Negative control clear?** | **Probe B proportion (%)** |
| --- | --- | --- | --- | --- |
| *Unaffected Controls* | | | | |
| Control 8 | 12/16 | No | Yes | 29.43 |
| Control 9 | 15/33 | No | Yes | 28.08 |
| Control 10 | 25/26 | No | Yes | 40.55 |
|  |  |  | Average | 36.02 ± 6.9 |
| *CTG18.1 expansion negative FECD* | | | | |
| FECD4exp- | 15/15 | No | Yes | 27.67 |
| FECD5exp- | 16/23 | No | Yes | 28.18 |
| FECD6exp- | 15/16 | No | Yes | 36.86 |
|  |  |  | Average | 30.90 ± 5.17 |
| *CTG18.1 expansion positive FECD* | | | | |
| FECD8exp+ | 63/88 | Yes | Yes | 15.05 |
| FECD9exp+ | 12/76 | Yes | Yes | 12.58 |
| FECD10exp+ | 18/67 | Yes | Yes | 21.36 |
|  |  |  | Average | 16.33 ± 4.53 |
