# Supplementary material for: Deciphering novel TCF4-driven mechanisms underlying a common triplet repeat expansion-mediated disease
Source: PLoS Genet. 2024 May 7;20(5):e1011230. doi: 10.1371/journal.pgen.1011230 (PMC11101122; doi:10.1371/journal.pgen.1011230)
Supplement: S16 Table — (DOCX) [file pgen.1011230.s019.docx]

**Table S16: *TCF4* isoform RNAScope sample summary with results of FISH with probe targeting repeat and negative RNAScope experiments (with probe targeting bacterial genes) in adult human dermal fibroblasts.**

| **Sample** | **Sample info** | **CTG18.1 repeat genotype** | **CUG**  **specific**  **foci present** | **Negative control clear?** | **Probe B proportion (%)** |
| --- | --- | --- | --- | --- | --- |
| *Unaffected Control Adult Human Dermal Fibroblast* | | | | | |
| Control HDF1 | F/62 | 22/27 | No | Yes | 42.83 |
| Control HDF2 | F/46 | 24/27 | No | Yes | 52.37 |
| Control HDF3 | F/38 | 12/15 | No | Yes | 28.30 |
|  | | |  | Average | 41.2 ± 12.1 |
| *CTG18.1 expansion positive FECD human dermal fibroblasts^a^* | | | | | |
| #F1*^a^* | F/69 | 31/69*^a^* | No*^a^* | Yes | 41.81 |
| #F2*^a^* | F/70 | 12/53*^a^* | No*^a^* | Yes | 37.8 |
| #F3*^a^* | F/65 | 84/108*^a^* | No*^a^* | Yes | 38.2 |
| #F4*^a^* | F/78 | 23/68*^a^* | No*^a^* | Yes | 48.3 |
| #F5*^a^* | M/82 | 23/70*^a^* | No*^a^* | Yes | 48.2 |
| #F6*^a^* | M/69 | 12/77*^a^* | No*^a^* | Yes | 50.9 |
|  | | | | Average | 44.2 ± 5.68 |
| *^a^*Data presented from Zarouchlioti et al 2018 | | | | | |
