# Supplementary material for: Deciphering novel TCF4-driven mechanisms underlying a common triplet repeat expansion-mediated disease
Source: PLoS Genet. 2024 May 7;20(5):e1011230. doi: 10.1371/journal.pgen.1011230 (PMC11101122; doi:10.1371/journal.pgen.1011230)
Supplement: S17 Table — (DOCX) [file pgen.1011230.s020.docx]

**Table S17:** Summary of rare, potentially deleterious, variants in FECD-associated genes identified in Proband B.

| **Variant (hg38)** | **Gene** | **HGVSc,  HGVSp** | **CADD, MAF gnomAD, MAF Kaviar** | **Proband** |
| --- | --- | --- | --- | --- |
| chr20-3231199-C-T | *SLC4A11* | Het c.1040G>A, p.(Arg347Gln) | 19.24, 1.39 × 10^-3^, 5.2 × 10^-4^ | Proband B 0/1 |
| Exome data from all probands with *TCF4* rare variants were interrogated to determine if rare and potentially deleterious variants in previously reported FECD-associated genes, including *COL8A2*, *SLC4A11*, *AGBL1*, *and ZEB1* could explain their respective disease (MAF < 0.005 in publicly available gnomAD genomes, exomes and Kaviar, CADD score > 15. A single heterozygous *SLC4A11* variant of uncertain significance was identified in Proband B. The annotation from Variant Effect Predictor is filtered to the “most severe” transcripts which is ENST00000380056.3. Abbreviations are as follows: Kaviar, Kaviar Genomic Variant database; gnomAD, The Genome Aggregation Database; UTR, untranslated region; MAF, minor allele frequency; HGVSc, coding DNA sequence based on the Human Genome Variation Society; HGVSp**,** protein sequence based on the Human Genome Variation Society. | | | | |
