# Supplementary material for: Deciphering novel TCF4-driven mechanisms underlying a common triplet repeat expansion-mediated disease
Source: PLoS Genet. 2024 May 7;20(5):e1011230. doi: 10.1371/journal.pgen.1011230 (PMC11101122; doi:10.1371/journal.pgen.1011230)
Supplement: S18 Table — (DOCX) [file pgen.1011230.s021.docx]

**Table S18:** A summary of FRAPOSA-derived ancestry information generated for all CTG18.1 Exp- using genome-wide SNP data extracted from exome sequencing data.

| **Predicted Ethnicity** | N **(%)** |
| --- | --- |
| AFR – African ancestry | 14/134 (10.4%) |
| EAS – East Asian ancestry | 1/134 (0.74%) |
| EUR – European ancestry | 119/134 (88.8%) |
