# Supplementary material for: Deciphering novel TCF4-driven mechanisms underlying a common triplet repeat expansion-mediated disease
Source: PLoS Genet. 2024 May 7;20(5):e1011230. doi: 10.1371/journal.pgen.1011230 (PMC11101122; doi:10.1371/journal.pgen.1011230)
Supplement: S19 Table — (DOCX) [file pgen.1011230.s022.docx]

**Table S19: Clinical data of probands with Fuchs endothelial corneal dystrophy identified to have rare and potentially deleterious heterozygous *TCF4* variants.**

| **Age***  **Sex**  **Ethnicity** | **CTG18.1 repeat genotype** | **Age at keratoplasty (years)***** | **CCT (μm)**  **before surgery** | **Pre-op**  **BCVA** | **CCT (μm)**  **after surgery** | **Final BCVA**** | **Associated ocular features** | **Family history (1º relatives)** |
| --- | --- | --- | --- | --- | --- | --- | --- | --- |
| 57/F/black  Proband A | 10/12 | 57 OD  59 OS | 495 OD  493 OS | NA | 441 OD  431 OS  (DMEK) | 0.67 OD  0.67 OS | Cataract surgery | Unknown |
| 42/M/black  Proband B | 17/23 | NP | 589 OD  577 OS | NP | NP | 0.67 OD  0.67 OS | Nil | Unknown |
| 61/F/mixed race (white/black)  Proband C | 13/18 | 61 OD  63 OS | NA  691 OS | NA | 681 OD (DSAEK)  539 OS (DMEK) | 1.0OD  0.67 OS | Cataract surgery | Unknown |
| 74/F/white  Proband D | 12/15 | 75 OD  74 OS | NA | NA | 442 OD  464 OS  (DMEK) | 1.0 OD  1.0 OS | Cataract surgery | Yes |
| 60/F/white  Proband E | 12/16 | 71 OD  60 OS  (PK) | 605 OD NA OS | 0.2 OD 0.25 OS | NA | 0.4 OD 0.6 OS | Cataract surgery,  secondary glaucoma | No |
| 64/F/white Proband F | 12/18 | 73 OD  72 OS | 640 OD  535 OS | 0.5 OD  0.5 OS | 572 OD  527 OS  (DMEK) | 0.67 OD  0.67 OS | Cataract surgery | Yes |
| 56/F/white Proband G | 12/18 | 59 OD  (PK)  63 OS  (DSAEK) 71 OS | NA | NA | 532 OD 539 OS | 0.5 OD 0.8 OS | EBMD, cataract surgery, secondary glaucoma | Yes |

M male, F female, OD right eye, OS left eye, DMEK Descemet membrane endothelial keratoplasty, DSAEK Descemet’s stripping automated endothelial keratoplasty, PK penetrating keratoplasty, CCT central corneal thickness, BCVA best corrected visual acuity, NP corneal surgery not performed, NA not available, EBMD epithelial basement membrane degeneration (Cogan),*Age at diagnosis, **BCVA values converted to decimal annotation, ***DMEK unless stated.
